# Supplementary material for: Conserved Evolution of MHC Supertypes among Japanese Frogs Suggests Selection for Bd Resistance
Source: Animals (Basel). 2023 Jun 27;13(13):2121. doi: 10.3390/ani13132121 (PMC10340028; doi:10.3390/ani13132121)
Supplement: Supplementary file 1 [file animals-13-02121-s001.zip › animals-2429889-supplementary.pdf]

Supplementary Tables and Figures

Table S1. Detailed summary of samples used for tissue collection to construct the transcriptome library. Transcriptome data for *Rana japonica, Rana ornativentris,* and *Rana tagoi tagoi* are from our previous study (Lau *et al.* 2017).

Species	Source	Sex	Date of capture/transfer to captivity
Bufo japonicus	Shiwa, Hiroshima 34°47′N, 132°67′E	M	2012/02/23
Dryophytes japonicus	Kurose, Hiroshima 34°33′N, 132°66′E	F	2004/07/02
Fejervarya kawamurai	Mt. Futagami, Hiroshima 34°40′N, 132°70′E	M	Not recorded
Glandirana rugosa	Takayama River, Hiroshima 34°53′N, 132°38′E	M	2011/07/14
Pelophylax nigromaculatus	Kurose, Hiroshima 34°33′N, 132°66′E	F	2009/07/02
Pelophylax porosus porosus	Kawashima-machi, Saitama 35°98′N, 139°49′E	F	2009/06/28
Buergeria buergeri	Uga, Hiroshima	F	2005/08/09
Buergeria japonica	Shirahama, Iriomote 24°37′N,123°77′E	M	Not recorded

Table S2. Summary of MHC class I supertypes and accession numbers. Bold and bold-italics indicate variants from Japanese frogs collected in our present study and Lau *et al.* (2016), respectively.

Supertype	MHC class I variants/alleles
ST-A	Agca-UA*02/04/05/11-14 [JQ679313/15/16/20/22-25]
	**Bfja -UA*02/03**
	Buga [KY302832/ KY302833]
	**Bubu**-UA*01
	**Drja-UA*04**
	Drja[#] [KY302810/11/16]
	Espr-UA*05/06/11/12 [JQ679335/36/41/42]
	**Feka-UA*04**
	**Glru-UA*01**
	Lica-UA*07/10/11 [JQ679349/52/53]
	Licl-UA*02-06/09/11/16 [JQ679356-60/63/65/70]
	Liya-UA*01/03/07/08
	Odma-UA*07-09 [MH784556-58]
	**Peni-UA*05**
	Poch-UA*05-07,09-10 [KX467508-10/12/13]
	Pome-UA*02/04/05/07 [KC261638/40/41/43]
	Psco [KX372230-34/38; KY072979]
	Rapi [AF185587.1]
	Rhom-UA*01-02/04-05/12-13/16-18 [KC261644/45/47/48/55/56/59-61]
	Smph-UA*01/02/04/06-11 [JQ679380/81/83/85-90]
ST-B	Agca-UA*08 [JQ679319]
	**Bubu-UA*02-05**
	**Buja_UA*01**
	Espr-UA*07 [JQ679337]
	**Feka-UA*01-03**
	**Glru-UA*02-05**
	Lica-UA*01-06/08/09/12 [JQ679343-48/50/51/54]
	Licl-UA*01/07/08/10/12/15 [JQ679355/61/62/64/66-69]
	Liya-UA*02/04-06/09
	Odma-UA*01-06 [MH784550-55]
	**Peni-UA*01-04**
	**Pepo-MHCI_UA*01-03**
	Poch-UA*08 [KX467511]
	Pome-UA*01/03/04/06 [KC261637/39/40/42]
	Psco [KX372241-42]
	Raja-UA*01-10
	Raor-UA*01-03/05-28
	Rata-UA*01-22
	Rhom-UA*03/06-11/14/15/19 [KC261646/49-54/57-58/62]
ST-C	Agca-UA*16-19 [JQ679327- JQ679330]
	Espr-UA*01 [JQ679331]
ST-D	Agca-UA*01/03/06/07/10 [JQ679312/14/17/18/21]
	**Bfja-UA*01**
	Buga [KY302834- KY302858]
	**Drja-UA*01-03,05-08**
	Drja[#] [KY302809/12-15/17-31]
	Espr-UA*02-04,08-10 [JQ679332-4,8-10]
	Smph-UA*03/05 [JQ679382/84]
	Xela [AF185580/83; NM_001085732]

[#]Korean population. Species that are reportedly susceptible to Bd are underlined. Species abbreviations: Agca - *Agalychnis callidryas*; Bfja - *Bufo japonicus*; Bubu - *Buergeria buergeri*; Buja - *Buergeria japonica*; Buga - *Bufo gargarizans*; Drja - *Dryophytes japonicus*; Espr - *Espadarana prosoblepon*; Feka - *Fejervarya kawamurai*; Glru - *Glandirana rugosa*; Lica - *Lithobates catesbeianus*; Licl - *Lithobates clamitans*; Odma - *Odorrana margaretae*; Peni - *Pelophylax nigromaculata*; Pepo - *Pelophylax porosus porosus*; Poch - *Rhacophorus chenfui*; Pome - *Polypedates megacephalus*; Psco - *Pseudophryne corroboree*; Raja - *Rana*

japonica; Raor - *Rana ornativentris*; Rata - *Rana tagoi tagoi*; Rapi - *Rana pipiens*; Rhom - *Zhangixalus omeimontis*; Smph – *Smilisca phaeota*; Xela - *Xenopus laevis*.

Table S3. Summary of MHC class II supertypes and accession numbers. Bold and bold-italics indicate variants from Japanese frogs collected in our present study and previous study (Lau *et al.* 2017), respectively.

Supertype	MHC class II variants/alleles
ST-1	**Bfja-01 – 03**
	Bfja [LC065651]
	Boor-05 [KJ679329]
	Buga-01 - 08 [KJ679317-24]
	**Buja-01/02**
	**Bubu-01/02**
	**Drja-01/04**
	Epca [HQ388288]
	**Feka-01/02**
	**Glru-01/02**
	Lica [BT081564; transcriptome data from DRA accession number SRP051787]
	Livea-1*/2*/3/3b*/11*/12/17/19/23 [KJ679288-91/304/05/10/12/16]
	Napa-DRB1*15 [XM_018558121]
	**Peni-03**
	**Pepo-01/02**
	Raja-01/02/04-06/08
	Raor-04/07/08/11
	Rata-01 -03/05 -11/13
	RhmaDAB01/DBB01/DCB01 [KP995700/04/06]
ST-2	Boor-02/06 [KJ679326/30]
	**Drja-02/03**
	Livea-4/6/7a/7b/9/10/15/18/20 [KJ679292/97/98/99/302/03/08/11/13]
	Raja-03/07/09
	Raor-01-03/05/06/09/10
	Rata-04/12
	Xetr [NP001039259], Xela [D13684, D13688]
ST-3	Rhom(Zhom) [KT276421/23/25/28/34/35/43/44/52/53/62/64/72/75/83/84]
	Ly1–32/40-42/79-80 [KU877031-62/70-72/107-108]
ST-4	Boor-01/03/04/07 [KJ679325/27/28/31]
	Livea-5a*/5b*/5c/5d/8a/8b/13*/14*/16/21/22 [KJ679293-96/300/01/06/07/09/14/15]
	Ly33-39/43-78 [KU877064-69/73-106]
	**Peni-01/02**
	Rhom [KT276499/505/08/10]
ST-5	Qusp [KJ082083]
	Rhom(Zhom) [KT276411-20/22/24/26/29-33/36-38/40/41/45-51/54-61/63/65-71/73/74/76-82/85-98/500-04/06/07/09/11/12]

Additional abbreviations: Qusp - *Quasipaa spinosa*, Boor - *Bombina orientalis*, Rhma - *Rhinella marina*, Napa - *Nanorana parkeri*, Livea - *Litoria verreauxii alpina*. Epca - *Epidalea calamita*. *MHC-II alleles from Livea suggested to be 'resistant' to Bd (Bataille *et al.* 2015). See Table S2 for other species abbreviations. Ly is equivalent to Liya [*Lithobates yavapaiensis*].

Table S4. List of peptides used for MHC binding prediction. A combined set of Bd-related proteins were analyzed (A), including three Bd-related antigens [70 kDa heat shock protein 3, 26S protease regulatory subunit 6A-B, and JEL423 surface antigen], and proteins of M36, S41, ASP, adhesion, CRN, and lipase3 genes with increased expression after Bd was inoculated in frog skin (Rosenblum *et al.* 2012). In addition, as a control, we examined (B) a set of 13 immunogenic antigens from human fugal pathogen, *Aspergillus fumigatus (*Bacher *et al.* 2014*)* (i.e. non-Bd-related antigens) and (C) a set of 7 antigens from *Escherichia coli* bacteria (i.e. non-fungal antigens). Full list of compiled peptide sequences (before and after protein cleavage) is available upon request.

Dataset/proteins	Accession number/ Locus tag	Original length (aa)	No. of 15-24 amino acid-long peptides after protein cleavage
**(A) Bd-related proteins and proteins with increased expression when Bd was grown in frog skin**			Total =644
70 kDa heat shock protein 3	XP_006675784	647	6
26S protease regulatory subunit 6A-B	XP_006680816	434	3
JEL423 surface antigen	OAJ39392	449	4
M36 (metallo-proteases)	BATDEDRAFT_11205	450	2
	BATDEDRAFT_1502	300	0
	BATDEDRAFT_16613	289	3
	BATDEDRAFT_1489	300	1
	BATDEDRAFT_1469	300	0
	BATDEDRAFT_12637	413	1
	BATDEDRAFT_1639	404	3
S41 (serine-proteases)	BATDEDRAFT_85649	883	11
	BATDEDRAFT_36653	100	0
	BATDEDRAFT_23534	640	6
	BATDEDRAFT_23310	883	12
	BATDEDRAFT_23544	705	8
	BATDEDRAFT_24207	843	9
	BATDEDRAFT_24208	322	3
	BATDEDRAFT_24156	885	11
	BATDEDRAFT_87928	571	8
	BATDEDRAFT_24985	928	10
	BATDEDRAFT_25462	629	6
	BATDEDRAFT_35365	885	10
	BATDEDRAFT_90146	883	10
	BATDEDRAFT_26287	643	6
	BATDEDRAFT_27937	882	8
	BATDEDRAFT_92476	577	8
ASP (aspartyl-proteases)	BATDEDRAFT_21660	480	5
	BATDEDRAFT_22611	503	5
	BATDEDRAFT_22623	476	3
	BATDEDRAFT_86720	475	4
	BATDEDRAFT_23192	491	5
	BATDEDRAFT_23275	525	6
	BATDEDRAFT_87177	423	5
	BATDEDRAFT_87185	476	8
	BATDEDRAFT_87250	475	10
	BATDEDRAFT_23765	503	7
	BATDEDRAFT_87859	389	3
	BATDEDRAFT_87892	472	7
	BATDEDRAFT_88273	481	4
	BATDEDRAFT_24300	476	7

	BATDEDRAFT_24380	492	6
	BATDEDRAFT_24760	490	5
	BATDEDRAFT_24767	487	3
	BATDEDRAFT_25148	477	4
	BATDEDRAFT_25666	491	6
	BATDEDRAFT_36999	302	3
	BATDEDRAFT_89345	469	6
	BATDEDRAFT_25259	509	6
	BATDEDRAFT_89380	431	5
	BATDEDRAFT_25355	541	7
	BATDEDRAFT_89821	477	2
	BATDEDRAFT_25680	384	6
	BATDEDRAFT_25784	418	2
	BATDEDRAFT_89959	458	5
	BATDEDRAFT_26088	379	5
	BATDEDRAFT_26151	476	6
	BATDEDRAFT_26132	482	9
	BATDEDRAFT_90236	179	2
	BATDEDRAFT_90237	241	3
	BATDEDRAFT_26134	476	4
	BATDEDRAFT_90267	476	3
	BATDEDRAFT_26425	477	7
	BATDEDRAFT_90625	478	4
	BATDEDRAFT_26741	477	5
	BATDEDRAFT_35725	441	6
	BATDEDRAFT_90888	375	4
	BATDEDRAFT_26762	473	9
	BATDEDRAFT_26758	464	5
	BATDEDRAFT_26748	452	5
	BATDEDRAFT_90978	400	6
	BATDEDRAFT_91075	405	7
	BATDEDRAFT_27277	475	7
	BATDEDRAFT_27069	484	3
	BATDEDRAFT_27286	399	7
	BATDEDRAFT_92020	471	5
	BATDEDRAFT_27966	488	5
	BATDEDRAFT_28166	469	5
	BATDEDRAFT_28328	487	8
	BATDEDRAFT_28256	490	4
	BATDEDRAFT_92488	442	6
	BATDEDRAFT_92592	475	2
	BATDEDRAFT_28513	478	5
	BATDEDRAFT_92740	212	4
	BATDEDRAFT_28541	559	7
	BATDEDRAFT_28537	400	6
	BATDEDRAFT_28684	484	9
Adhesin genes	BATDEDRAFT_84886	149	0
	BATDEDRAFT_22355	363	5
	BATDEDRAFT_21697	569	4
	BATDEDRAFT_85835	303	2
	BATDEDRAFT_85893	302	6
	BATDEDRAFT_22768	376	0
	BATDEDRAFT_87248	379	6
	BATDEDRAFT_87422	284	1
	BATDEDRAFT_23878	345	6
	BATDEDRAFT_87864	324	3
	BATDEDRAFT_87929	340	1
	BATDEDRAFT_11071	328	3
	BATDEDRAFT_90520	323	6

	BATDEDRAFT_27092	457	0
	BATDEDRAFT_27091	663	3
	BATDEDRAFT_28554	993	9
	BATDEDRAFT_93124	563	2
CRN (crinkler-like effectors)	BATDEDRAFT_84882	596	6
	BATDEDRAFT_85109	603	6
	BATDEDRAFT_86517	222	1
	BATDEDRAFT_31422	673	9
	BATDEDRAFT_23217	281	1
	BATDEDRAFT_87221	806	9
	BATDEDRAFT_87524	769	8
	BATDEDRAFT_24811	671	6
	BATDEDRAFT_37012	765	9
	BATDEDRAFT_26085	458	4
	BATDEDRAFT_26137	671	7
	BATDEDRAFT_90343	561	6
	BATDEDRAFT_90726	354	2
	BATDEDRAFT_26749	780	8
	BATDEDRAFT_28183	721	8
Lipase-3 encoding genes	BATDEDRAFT_86691	407	4
	BATDEDRAFT_86693	323	3
	BATDEDRAFT_89307	261	2
	BATDEDRAFT_26489	250	2
	BATDEDRAFT_26490	376	6
	BATDEDRAFT_26491	470	7
	BATDEDRAFT_93190	284	3
	BATDEDRAFT_93191	375	3
**(B) Single *A. fumigatus* proteins**			Total: 49
Scw4 (Cell wall glucanase)	AFUA_6G12380	369	2
Gel1 (1,3-β-glucanosyltransferase)	AFUA_2G01170	452	7
Crf1/Aspf9 (Cell wall glucanase)	AFUA_1G16190	395	2
Pst1 (GPI-anchored cell wall protein)	AFUA_6G10290	406	8
Sod3/Aspf6 (Manganese superoxide dismutase)	AFUA_1G14550	210	3
Shm2 (Serine hydroxylmethyltransferase)	AFUA_3G09320	471	2
Aspf2 (Allergen, expressed in zinc-limiting	AFUA_4G09580	310	3
conditions)	AFUA_4G13170	316	5
CpcB (RACK1 ortholog)	AFUA_5G13450	256	3
TpiA (Triosephosphate isomerase)	AFUA_6G02280	438	3
Aspf3 (Peroxiredoxin family reductase)	AFUA_6G09740	334	3
GliT (Gliotoxin oxidase)	AFUA_3G02270	728	3
CatB (Catalase B)	AFUA_1G04130	307	5
FG-GAP (FG-GAP repeat protein)			
**(C) *E. coli* proteins**			Total: 24
Surface antigen	SYX50777.1	179	1
clpG surface antigen	AAA23585.1	278	1
Surface antigen	EFX23024.1	577	7
Capsular polysaccharide	ALY13557.1	540	8
Capsular polysaccharide	GCO29334.1	248	4
Lipopolysaccharide 1,2-glucosyltransferase RfaJ	WP_032226820.1	338	3

Table S5. Nucleotide lengths of mitochondrial gene sequence collated from published and transcriptomic data. These sequences were concatenated to generate the species phylogeny shown in Figure 3.

Species name	GenBank ID of mitochondrial genome	Nucleotide length												Concatenated nucleotide length
		ND1	ND3	ND4	ND4L	ND5	ND6	COX1	COX2	COX3	ATP6	ATP8	CYTB	
Alytes obstetricans pertinax	NC_006688.1	960	342	1377	294	1806	507	1548	687	783	681	165	1140	10290
Andrias davidianus	NC_004926.1	969	348	1374	294	1809	516	1548	687	783	681	165	1140	10314
Ascaphus truei	AJ871087.1	969	348	1371	294	1800	507	1548	687	783	681	165	1140	10293
Bombina fortinuptialis	NC_006402.1	960	342	1377	294	1806	507	1551	687	783	681	165	1140	10293
Bombina maxima	NC_011049.1	960	339	1377	294	1806	546	1551	687	783	681	165	1140	10329
Bombina orientalis	NC_006689.1	960	342	1377	294	1806	507	1551	687	783	681	165	1140	10293
Bombina variegata	NC_009258.1	960	342	1377	294	1806	507	1551	687	783	681	165	1140	10293
Buergeria buergeri	AB127977.1	963	342	1365	282	1788	489	1548	684	783	681	162	1167	10254
Bufo gargarizans	NC_008410.1	960	339	1362	297	1788	492	1539	687	783	681	162	1143	10233
Bufo melanostictus	NC_005794.2	960	339	1362	297	1788	492	1539	687	783	681	162	1143	10233
Discoglossus galganoi	NC_006690.1	963	342	1377	294	1815	507	1551	687	783	681	165	1140	10305
Euphlyctis hexadactylus	NC_014584.1	960	339	1353	279	1845	483	1548	678	783	681	159	1140	10248
Fejervarya cancrivora	NC_012647.1	954	342	1359	276	1803	486	1533	681	783	681	162	1134	10194
Fejervarya limnocharis	NC_005055.1	957	342	1350	276	1800	486	1533	681	783	681	159	1134	10182
Glandirana emeljanovi	KF771343.1	960	339	1359	282	1788	495	1548	687	783	681	162	1140	10224
Glandirana rugosa	KF771341.1	960	339	1359	282	1788	495	1548	687	783	681	162	1140	10224
Glandirana tientaiensis	KF771342.1	960	339	1359	282	1788	492	1548	687	783	681	162	1140	10221
Bufo japonicus	NC_009886.1	960	339	1362	297	1800	492	1539	687	783	681	162	1143	10245
Hoplobatrachus tigerinus	NC_014581.1	960	339	1356	279	1824	483	1548	681	783	681	159	1143	10236
Hymenochirus boettgeri	NC_015615.1	966	342	1377	294	1815	513	1545	687	783	657	165	1140	10284
Hyla chinensis	NC_006403.1	960	339	1362	300	1788	495	1539	687	783	681	162	1146	10242
Dryophytes japonicus	NC_010232.1	960	339	1362	300	1800	495	1539	687	783	681	162	1146	10254
Kaloula pulchra	NC_006405.1	957	339	1359	282	1791	501	1548	687	783	681	162	1140	10230
Leiopelma archeyi	NC_014691.1	966	348	1377	294	1824	507	1548	687	783	681	162	1140	10317
Limnonectes fujianensis	AY974191.2	954	324	1365	282	1824	489	1536	684	783	681	159	1239	10320
Mantella madagascariensis	NC_007888.1	960	339	1362	279	1794	486	1548	687	783	681	162	1140	10221
Microhyla heymonsi	NC_006406.1	957	339	1359	282	1761	498	1548	687	783	681	162	1143	10200
Microhyla okinavensis	NC_010233.1	957	339	1362	282	1803	498	1548	687	783	681	162	1140	10242
Microhyla ornata	NC_009422.1	957	339	1362	282	1791	501	1548	687	783	681	162	1143	10236
Nanorana pleskei	NC_016119.1	957	357	1362	282	1821	495	1548	684	783	681	159	1143	10272
Occidozyga martensii	NC_014685.1	960	342	1365	276	1806	489	1545	687	783	681	159	1134	10227
Odorrana ishikawae	NC_015305.1	960	339	1365	282	1800	498	1548	687	783	675	165	1137	10239
Amolops tormotus	NC_009423.1	960	309	1365	282	1788	498	1551	687	783	675	165	1143	10206
Pelophylax chosenicus	NC_016059.1	960	339	1359	282	1791	498	1536	687	783	681	162	1140	10218
Pelobates cultripes	AJ871086.1	972	342	1377	294	1818	507	1551	681	783	681	165	1140	10311
Pelophylax nigromaculatus	NC_002805.1	960	339	1359	282	1794	498	1536	687	783	681	162	1140	10221
Pelophylax plancyi	NC_009264.1	960	339	1359	282	1794	498	1536	687	783	681	162	1140	10221

Pipa carvalhoi	NC_015617.1	963	342	1377	294	1809	510	1548	687	783	681	165	1140	10299
Pipa pipa	GQ244477.1	963	342	1377	294	1809	504	1545	687	783	681	165	1140	10290
Pseudhymenochirus merlini	NC_015618.1	963	342	1374	294	1809	516	1545	684	783	681	165	1137	10293
Quasipaa spinosa	NC_013270.1	954	339	1362	282	1821	495	1548	684	783	681	159	1143	10251
Rana adenopleura	JX033120.1	960	339	1362	282	1797	498	1548	687	783	675	162	1140	10233
Lithobates catesbeianus	KF049927.1	960	339	1359	282	1809	498	1551	687	783	678	159	1140	10245
Rana kunyuensis	KF840516.1	957	339	1359	282	1809	492	1551	684	783	681	159	1140	10236
Rana dybowskii	KF898355.1	957	339	1359	282	1791	492	1548	687	783	681	159	1140	10218
Rana sylvatica	KP222281.1	960	339	1359	282	1791	498	1551	687	783	681	159	1140	10230
Rhinophrynus dorsalis	NC_015620.1	963	342	1377	294	1821	507	1551	687	783	681	165	1140	10311
Rhacophorus schlegelii	NC_007178.1	960	342	1362	282	1794	486	1551	681	783	621	162	1143	10167
Xenopus laevis	NC_001573.1	969	342	1383	294	1812	510	1554	687	780	678	165	1137	10311
Xenopus tropicalis	NC_006839.1	966	342	1377	294	1815	516	1554	687	783	681	165	1140	10320
**PRESENT STUDY:**														
Bufo japonicus		969	342	1374	300	1798	495	1555	688	786	684	165	1143	10299
Fejervarya kawamurai		960	342	1362	279	1820	**363**	1548	682	786	684	165	1139	10130
Hyla japonica		969	339	1374	303	1812	498	1554	688	786	684	165	1143	10315
Glandirana rugosa		966	342	1370	285	1798	498	1558	**495**	**626**	684	165	**1036**	9823
Rana japonica		966	342	1368	294	1789	495	1557	**572**	772	684	165	**705**	9709
Rana ornativentris		966	342	1368	294	1800	495	1551	688	786	**524**	165	1143	10122
Rana tagoi tagoi		966	342	1364	294	1792	495	1557	**657**	786	684	165	1143	10245
Pelophylax nigromaculatus		966	342	**1345**	285	1792	501	**1040**	688	786	684	165	1142	9736
Pelophylax porosa		969	342	1353	300	1798	495	1554	688	786	684	165	1143	10277
Buergeria buergeri		960	342	1369	285	1800	492	1557	688	786	684	165	1143	10271
Buergeria japonica		960	342	1372	279	1789	492	1525	688	786	**606**	165	1141	10145

Species names in bold indicate overlap between publically available mitochondrial genome sequences and the present study. Nucleotide lengths in bold indicate incompletely mapped genes from our transcriptome data.

Table S6. Chi-square analyses of MHC class I supertypes and assumed/reported Bd susceptibility.

	Observed no. of alleles			
MHC-I supertype	Susceptible	Tolerant	Resistant	Total
ST-A	11	32	35	78
ST-B	7	19	100	126
ST-C	0	5	0	5
ST-D	0	13	56	69
Total	18	69	191	278
	Expected no. of alleles			
MHC-I supertype	Susceptible	Tolerant	Resistant	Total
ST-A	5.05	19.36	53.59	78
ST-B	8.16	31.27	86.57	126
ST-C	0.32	1.24	3.44	5
ST-D	4.47	17.13	47.41	69
Total	18	69	191	278
**Chisq p-value**	**3.044E-09**			

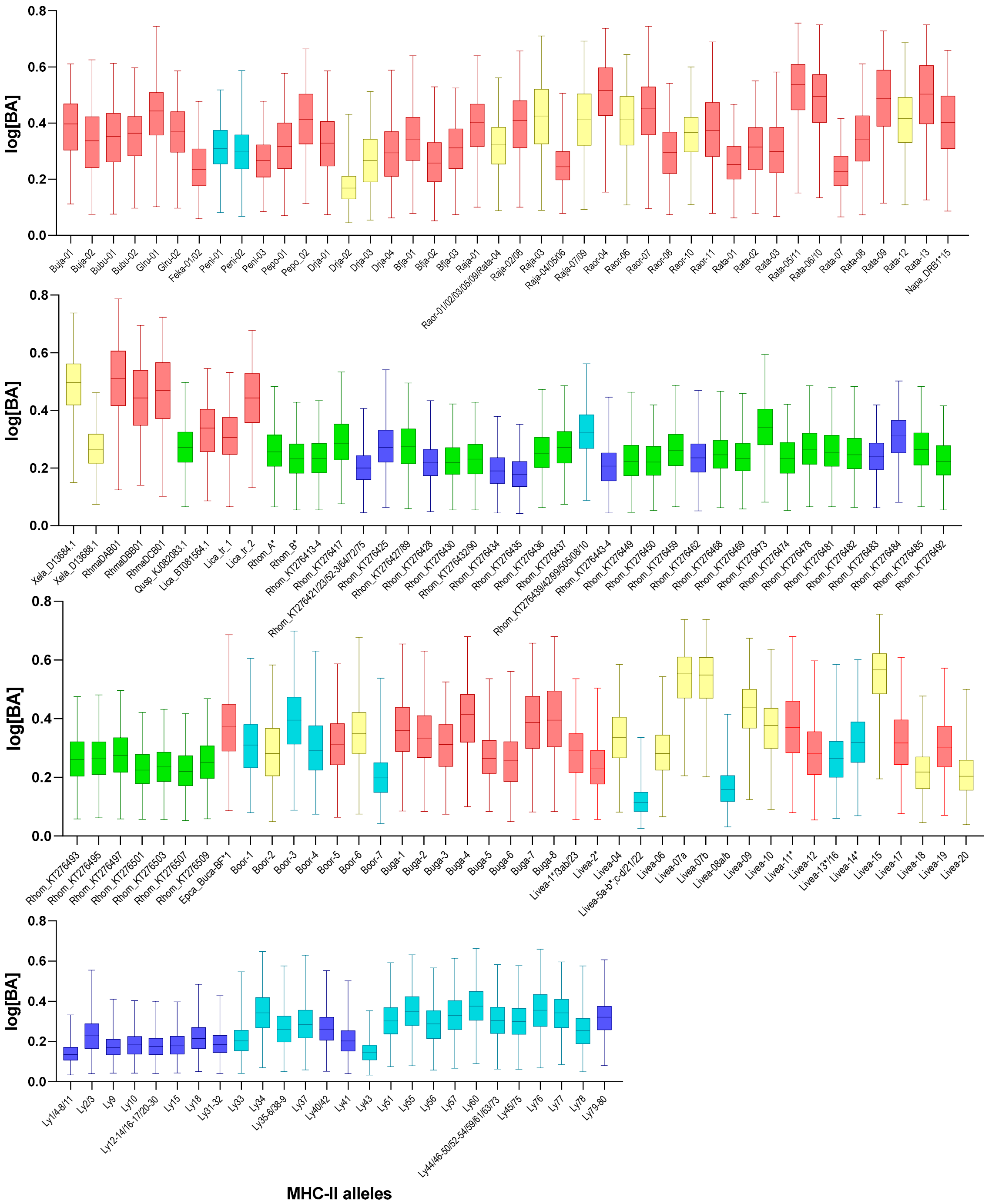

Figure S1. Binding affinity scores (log[BA]) of each MHC class II allele or variant against the Bd-related peptides. Whiskers indicate maximum and minimum log[BA] scores. MHC-II supertypes are indicated by colours: ST-1: red; ST-2: yellow; ST-3: navy; ST-4: green; ST-5: light blue.

** Livea MHC-II alleles suggested to be 'resistant' to Bd* (Bataille *et al.* 2015)

A: Rhom_KT276411/13-14/16/18-20/22/26/38/40-1/45-48/51/54-8/60-1/63/65-7/70-1/76/80/94/KT276512*

B: Rhom_KT276412/24/29/31/33/77/79/86-8/91/96/99/ KT276500/02/04/06/11*
